# Providing LGBTQ+ affirming care to young adults with inflammatory bowel diseases

**DOI:** 10.1016/j.hctj.2023.100030

**Published:** 2023-11-30

**Authors:** Cade Johnson, Sneha Dave, Sydney Reed, Victor Chedid, Lucy Laube, Laura Targownik

**Affiliations:** a Generation Patient, Crohn’s and Colitis Young Adults Network, United States; b Division of Gastroenterology and Hepatology, Director of IBD Pride Clinic, Mayo Clinic, Rochester, MN, United States; c National Psoriasis Foundation, Crohn’s and Colitis Young Adults Network, United States; d Departmental Division Director (Gastroenterology and Hepatology), University of Toronto, Past Chair, Diversity and Equity, Canadian Association of Gastroenterology, Staff Gastroenterologist, Mount Sinai Hospital (Toronto), Canada

**Keywords:** Young adults with inflammatory bowel diseases, Adolescents with inflammatory bowel disease, LGBTQ+ and inflammatory bowel disease, LGBTQ+ youth with inflammatory bowel disease

## Abstract

Creating an affirming care environment for young adults with IBD in the LGBTQ+ community is an essential part of inflammatory bowel disease (IBD) care. This article summarizes the discussions held during the Roundtable on Young Adults with IBD, which focused on essential information for adult-care providers to successfully navigate the complexities and intricacies of sexuality and gender identity for young adult IBD patients. The Roundtable on Young Adults with IBD is held through the Crohn’s and Colitis Young Adults Network. Key focus areas include establishing effective communication between providers and patients, maintaining patient safety and privacy, emphasizing trauma-informed practices, and building trust-based provider-patient relationships. Addressing these issues will allow providers to more fully and effectively treat LGBTQ+ young adults with IBD, ensuring a better, safer path into successful adult lives.

## Introduction

The Roundtable on Young Adults with Inflammatory Bowel Disease (IBD) is a year-long learning community of monthly discussions between patients and providers striving to improve outcomes for young adult IBD patients. Each monthly discussion focuses on a pressing issue among this patient population, with our fifth roundtable concentrating on the LGBTQ+ community and associated issues. Overarching themes that permeated this discussion centered upon the pressing need for practical, trust-based communication and relationships between LGBTQ+ young adult patients and providers. The LGBTQ+ community represents a significant–and likely growing–portion of the youth population. According to the GLAAD 2017 Accelerating Acceptance survey, 20% of respondents aged 18–34 identify as LGBTQ+ compared to 12% of the total U.S. population.^1^ While the transition into adult life is a vulnerable period for all young patients with IBD, this is especially true for LGBTQ+ youth, who have concerns and risks specific to their experiences and identities. These may include but are not limited to access to trauma-informed and gender-affirming care as well as physicians with adequate knowledge of appropriate medical care for transgender and gender-nonconforming individuals with IBD, particularly as it relates to medications, surgeries, and mental health. Furthermore, stress management and support systems are key for the successful management of IBD, especially during the transition from pediatric to adult care, and thus, special attention is required from providers when treating young adults in the LGBTQ+ community.

## Importance of inclusive communication for comprehensive care

Provider questionnaires are the first step in the assessment process for patients, whether for those in acute distress in emergency facilities or those seeking maintenance care for their IBD. It is crucial that authors of these questions carefully consider word choice, tone, and context. When speaking about visiting the emergency room with her female partner, a lesbian-identified young adult IBD patient reported feelings of frustration and embarrassment when providers repeatedly asked if her severe abdominal pain could be the result of pregnancy. This questioning went far beyond necessary diagnostic inquiry and actively prevented a timely response to the issues at hand with her IBD. This unwarranted delay in treatment only served to subject the patient to additional stress. An adult provider added that the “disease scripts” taught in medical training are typically structured around “one-liners” that are not often relevant to treating IBD and are based on assumptions about gender identity and sexual orientation. The provider gave an example in that men who have sex with men (often abbreviated to MSM, including gay, bisexual, and queer-identified men) are sometimes stereotyped by medical providers as being especially likely to have STIs. While there is a greater prevalence of certain STIs in MSM communities, that does not mean that it should be the primary diagnostic option or that it should be suspected over IBD in all MSM.^2^ This is especially true given recent research that has shed light on the elevated prevalence of IBD within MSM communities.^3^

## Emphasizing trauma-informed best practices of care

The LGBTQ+ population, in general, is disproportionately at risk for interpersonal, sexual, and identity-based violence, with LGBTQ+ youth, transgender and gender-expansive youth, and LGBTQ+ youth of color even more so at risk compared to peers.^4^ Providers must be aware that young adults in the LGBTQ+ community have a significant likelihood of having already experienced sexual violence, whether that be child sexual abuse or partner-based violence.^2^ As IBD treatment and gastrointestinal treatment in general often require close contact between providers and intimate areas of a patient’s body, such as the genitals or anal region, trauma-informed practices are key not only to avoid re-traumatization but also to ensure the most effective care of IBD. An adult provider emphasized that successful care of IBD requires a “full patient” approach, meaning that physical symptoms cannot be treated in isolation from the life experiences of the patient as a unique individual, including adverse life experiences. Additionally, providers should avoid making assumptions about support systems and care providers for young adult patients in the LGBTQ+ community. LGBTQ+ youth are more likely to be estranged from biological family members and traditional support systems, relying on friends, roommates, teachers, and other community members in their stead.^5^ Conversely, many LGBTQ+ youth, particularly youth of color, immigrant youth, and youth from minority religious groups, may not be “out” to everyone in their lives and might only be comfortable discussing their LGBTQ+ identities in certain contexts.^5^ This is especially crucial when considering the well-being and safety of LGBTQ+ young adults with IBD, as severe chronic conditions like IBD may require them to rely on care from family or community members who may not be accepting of queer identities.

## Considerations for patient privacy and safety

Both adult providers and young adult patients recognized challenges in collecting information on patient gender and sexual identity. One adult provider underlined the importance of avoiding putting the onus on a patient to self-disclose, as many LGBTQ+ people have learned that doing so is not always safe, even in clinical settings. Instead, healthcare systems should create affirming structures. Even as individual practitioners may endeavor to be inclusive, efforts by a single person do not replace larger systemic inequities. A young adult patient added that giving context as to why questions of gender and sexual identity are being asked is key—sharing information and context as to how diagnosing and treating IBD functions as a provider performs a dual function. It involves young adult patients in their care, assisting them to be empowered as self-advocates and assuring them that questions are being asked to provide comprehensive care. Unfortunately, the recent uptick of anti-LGBTQ+ legislation and policy in certain U.S. states can also cause a “chilling effect” even to young adult patients living in unaffected jurisdictions, creating additional stress and reluctance to self-disclose.^6^ Fear of mistreatment by medical providers is a significant barrier for trans individuals seeking care, with 23% of transgender individuals surveyed reporting avoiding medical care for this reason. This fear is not irrational, with 33% of transgender individuals who do seek care reporting a negative experience.^7^ However, there are proposed solutions to this issue. Of those respondents who had negative experiences, most indicated a need for providers to be educated on transgender and gender-nonconforming care by patients themselves during appointments.^7^ Self-education by providers and a proactive approach with patients would make a significant difference in mitigating this issue of self-selecting out of care.

Furthermore, there is a shortage of research on transgender and nonconforming people with IBD, with no existing current guidelines on treatment. Research is especially needed on interactions between IBD and gender-affirming medical care. One example is that certain types of vaginoplasty surgery utilize tissue from the bowels, which, for young adult patients with IBD, creates a long-term need for different screenings for colorectal cancers as well as education both for their providers and the patients themselves.^8^

## Creating trust-based patient-provider relationships

Though there are significant barriers and challenges in treating young adult patients with IBD in the LGBTQ+ community, there are a variety of strategies and actions providers can take to create an affirming care environment. One such approach, as suggested by a young adult patient, is to display the progress pride flag in prominent areas of the office, particularly in the reception and examination rooms. Small symbols indicating that providers are committed to creating safe spaces can make big differences in patient comfort, particularly for young adults transitioning out of pediatric care who might be attending medical appointments on their own for the first time. An adult provider also mentioned that for transgender and gender-diverse patients in particular, providers could underline their understanding that transgender care, such as hormone treatment and gender-affirming surgeries, are not “cosmetic” or necessarily secondary to other forms of medical care. If the importance of this issue is not fully understood by providers or communicated to patients, there is a risk of compromising crucial trust in the relationship. However, affirming support and acceptance can make a meaningful difference in engendering trust, reducing stress during clinical appointments, and facilitating more effective, open communication.

## Conclusion

This discussion on providing affirming care to ult patients was held as part of the Roundtable on Young Adults with IBD. Throughout the conversation, participants made up of both healthcare providers and young adult patients with IBD, highlighted the need for effective communication, patient safety, trauma-informed practices, and trust-based relationships. These considerations are essential to ensure that LGBTQ+ young adults with IBD receive comprehensive care, allowing them to transition into adulthood with better health outcomes.

While challenges exist, such as the need for more research and guidelines for transgender individuals with IBD, healthcare providers can take tangible steps to create affirming care environments. Displaying symbols of support and recognizing the significance of transgender care can foster trust and open communication. In summary, by prioritizing LGBTQ+ -affirming care, healthcare providers can play a pivotal role in enhancing the well-being of young adult patients with IBD in the LGBTQ+ community as they embark on their journey into adulthood.

## Human rights

This article does not contain any studies with human participants performed by any of the authors.

## Welfare of animals

This article does not contain any studies with animals performed by any of the authors.

## Funding

This work was supported by The Leona M. and Harry B. 10.13039/100007028Helmsley Charitable Trust.

## Declaration of Competing Interest

None.

## Data Availability

No data was used for the research described in the article.
